# Faculty readiness for online teaching at Imam Abdulrahman Bin Faisal University during the COVID-19 crisis: a cross-sectional study

**DOI:** 10.12688/f1000research.28173.1

**Published:** 2021-08-20

**Authors:** Muneerah B. Almahasheer, Abdullah Al Rubaish, Abdullah Alkadi, Mahmoud A. Abdellatif, Vijaya Ravinayagam, Assaf, Wael Fateh, Palanivel Rubavathi Marimuthu, Nuhad A. Alomair

**Affiliations:** 1 Department of English, College of Arts, Imam Abdulrahman Bin Faisal University, Dammam, 31441, Saudi Arabia; 2 Deanship of eLearning & Distance Learning, Imam Abdulrahman BinFaisal University, Dammam, 31441, Saudi Arabia; 3 College of Medicine, Imam Abdulrahman Bin Faisal University, Dammam, 31441, Saudi Arabia; 4 College of Architecture and Planning, Imam Abdulrahman BinFaisal University, Dammam, 31441, Saudi Arabia; 5 Deanship of Scientific Research & Institute for Research and Medical Consultations, Imam Abdulrahman BinFaisal University, Dammam, 31441, Saudi Arabia; 6 Deanship of Quality and Academic Accreditation, Imam Abdulrahman BinFaisal University, Dammam, 31441, Saudi Arabia; 7 Deanship of Scientific Research, Imam Abdulrahman Bin Faisal University, Dammam, 31441, Saudi Arabia; 8 Department of Chemistry, Imam Abdulrahman BinFaisal University, Dammam, 31441, Saudi Arabia

**Keywords:** COVID-19, Education, Faculty readiness, Administration, Remote working.

## Abstract

**Background:** The outbreak of the COVID-19 pandemic has affected the education sector around the world. In order to control the spread of the virus, eLearning practice has been introduced in Saudi higher education. Such online communication has become an important tool to narrow the teaching practice gap. This study assessed the characteristics of eLearning and distance learning and the inclination of Imam Abdulrahman BinFaisal University (IAU) faculty members in terms of skills, and managing classes and tests using online learning tools.

**Methods:** A QuestionPro questionnaire with 22 questions on eLearning experience, training experience, and skills and knowledge in the educational process of IAU teaching faculty was conducted through the online university e-mail domain. The questionnaire was sent to the IAU’s teaching faculty.

The questionnaire’s reliability was studied using Cronbach’s α coefficient. The criterion value was statistically studied using the KMO (Kaiser-Meyer-Olkin) and Bartlett’s test. The variables associated with the present survey model were analysed using Structural Equation Modelling (SEM).

**Results:** The study showed positive responses and readiness (skills and abilities) and the effectiveness of IAU’s faculty members to perform e-learning activities during COVID-19. IAU faculty received a strong positive response, and the respondents were also impressed with and agreed on trainer knowledge, session management, communication and expertise on training topics.

**Conclusions:** The positive response indicates the readiness of IAU to provide the necessary support (tools, information and updates) required for a successful online educational process.

## Introduction

Coronavirus disease 2019 (COVID-19) derived from a mutated zoonotic virus tends to cause respiratory infections with variable severity ( Tay *et al.*, 2020). The pandemic disease initially started in Wuhan city, China and then spread severely, affecting Western countries. The infection rate and number of deaths are increasing at alarming rates around the globe. The main source of this rapid spread is attributed to the infection and replication ability of the virus in the upper respiratory tract without any symptoms. In the case of a single cough or sneeze, millions of microdroplets containing the virus are generated. COVID-19 has infected approximately 16 million people and caused the death of approximately 600,000 individuals worldwide. The global economic losses due to forced lockdowns around the globe to contain the spread of the virus have been estimated to be 9 trillion US dollars. In the U.S., more than 4 million people have been infected; and in the Kingdom of Saudi Arabia (KSA), the cases of infection and deaths are increasing steadily. The transmission rate from infected people was found to be higher than that of the influenza virus with reproductive numbers between 1.4 and 2.5. In the KSA, to date (08.04.21), the total number of cases is 394,952 with 6,719 deaths and 381,189 recoveries ( https://www.worldometers.info/coronavirus/).

To contain the spread of this viral infection, strict social distancing, quarantine and rapid testing are suggested to control the COVID-19 crisis ( Giordano *et al.*, 2020). Inadvertently, the important role of information technology has been felt in higher education ( Ayers, 2004; Carr-Chellman & Duchastel, 2000). Saudi Arabia has initiated several measures to actively control and manage the virus. In order to fight against COVID-19, as per Saudi government instructions, the Ministry of Education has taken several actions without affecting the quality of the education system.

Imam Abdulrahman Bin Faisal University (IAU) is a leading university promoting academic and advanced scientific research in the Eastern Region. IAU has various graduate courses and branches. The university started with the College of Medicine and College of Architecture and provides strong health care services through the establishment of King Fahd University Hospital. The IAU campus and its 21 colleges spread across various places of the Eastern Province with student enrolment is currently approximately 45,000 students. In order to control the spread of COVID-19, the KSA suspended all onsite activities of universities and initiated digital-based distance learning and remote working strategies. Based on the World Health Organization and Ministry of Health guidance, certain orders were issued such as staying home, working from home, being safe, and maintaining good hygiene. In the case when going out is a necessity, social distancing (2 m) should be maintained. The sudden health crisis affected the educational sectors and inflicted a long-term financial revision state pertaining to online education.

Web-based advanced learning tools for online teaching have long been considered a prime importance for student coaching ( Beaudoin, 1990; Beaudoin 1998; Cohn, 2002; Zhang *et al.*, 2020). Therefore, in response to government mandated quarantine and remote working, IAU swiftly moved to online teaching (March, 2020). Fortunately, various technological updated measures have already been recommended based on the KSA’s Vision 2030 ( https://ndu.gov.sa/en/). Accordingly, several technological readiness measurements have been implemented by IAU. The advancement of the digital age with computer-based information technology was well realized, leading to the establishment of the Deanship of E-Learning and Distance Learning in 2010. Since then, the evolution of the eLearning processes of universities worldwide has been constantly upgraded and developed for teaching, distance learning programmes, training and services ( Dhawan, 2020). The IAU’s mission to integrate such digital technology aims to provide effective eLearning teaching, provide distance learning services and deliver support in e-courses. The goal is to provide an e-learning platform to on- and off-campus students and expand the technology from universities to integrate regions and spread across the KSA.

Currently, the digital platform that IAU uses is the Blackboard eLearning management system. The Deanship of E-Learning and Distant Learning lab at IAU is integrated with advanced high-performance IOS computers (Mac), Windows, platforms, visual viewers, studios, and soundproof capsules (to view and recording services). The presence of a digital lab enables interactive sessions, displays, video meetings, lectures, workshops, training sessions, uploading data in the Blackboard system and recording. IAU has advanced eLearning digital management facilities.

The aim of this study was to analyse the significant role of the digital system and evaluate the readiness of IAU faculty members to transition to online teaching during COVID-19 using survey-based methods.

## Methods

### Study design

The questionnaire was intended to study the level of eLearning experience among the faculty members of IAU. Considering the abrupt changes in teaching mode during this pandemic situation, a questionnaire could effectively predict the characteristics and management of the advantages of e-learning by faculty members. The study was conducted from 8 ^th^ March to 12 ^th^ March 2020.

### Ethical considerations

The study was approved by the Institutional Review Board (Standing Committee for Research Ethics on Living Creatures) with reference no. IRB-2020-17-148). Completion of the questionnaire by faculty members was taken as consent to participate.

### Participants

This study was conducted at Imam Abdulrahman Bin Faisal University (IAU), which is located in the Eastern Region of Saudi Arabia. All faculty members (N=2227) of IAU who were involved in online teaching during the COVID-19 crisis in the 2019–2020 academic year were considered the population of this study, as only these faculty members used and experienced IAU e-learning facilities. Access to QuestionPro by external (non-IAU) persons was prohibited; therefore, only IAU teaching faculty were included. Nonteaching staff/faculty of IAU were excluded. In order to address potential sources of bias, the population of this study only included faculty members.

### Data collection

A QuestionPro questionnaire with 22 questions on eLearning experience, training experience and skills and knowledge in the educational process of IAU faculty was implemented. Questionnaire was sent to participants using their university e-mail with a link to the questionnaire. The faculty members had to use their university email and password to log into the questionnaire via Blackboard dashboard. A specified time duration of 14 days to respond to the questionnaire was given to potential respondents. Two follow-up emails were sent that included reminders regarding answering the questionnaire.

The questionnaire was created through four brainstorming meetings with higher education experts and faculty members.

Three sections were included in the questionnaire, which aimed to evaluate: (section 1) the overall eLearning experience using Blackboard; (section 2) the skills and training provided to IAU faculty members to use eLearning; and (section 3) the management of classes and tests using the online learning tools. Section 1 had 8 items, section 2 had 9 items and section 3 had 4 items (total 21 items). The last item (22) was ‘How satisfied are you with our services’. Each item was a statement, and the answers respondents could choose from were as follows: strongly agree (marked as 1 in the data), agree (2), true sometimes (3), disagree (4), and strongly disagree (5).

### Data analysis

Descriptive statistics were applied to reveal the level of eLearning experience among the faculty members of IAU. The internal consistency of the questionnaire was assessed using Cronbach’s alpha reliability test. Confirmatory factor analysis (CFA) with the principal component method was used to determine the construct validity of the questionnaire used. Furthermore, structural equation modelling (SEM) analysis was conducted using the AMOS (Analysis of Moment Structures) software 2020 to study the adequacy of the e-learning variables involved in the questionnaire. Pearson’s correlation was also used to examine the relationship between the e-learning variables and the faculty’s overall satisfaction. Besides, the effect of e-learning variables on the faculty’s overall satisfaction was evaluated using multiple regression analysis. All statistical analyses were conducted using SPSS version 22.0 at a 5% significance level. Pearson’s correlation was also used to examine the relationship between the e-learning subgroups and interactions. There were no missing data to address in this study.

## Results

Out of the 2227 potential responses, 634 completed responses were received (response rate, 28.5%).

### Reliability and validity of the instrument

Cronbach’s alpha was used as a benchmark to study the reliability of the questionnaire ( Schakib-Ekbatan *et al.*, 2019). The reliability value of Cronbach’s α coefficient ranges from 0.00–1.00. In the present study, the reliability of the statistics on the eLearning questionnaire using Cronbach’s α coefficient was found to be 0.940. This indicates that the questionnaire achieved a reliable standard of high consistency.

Faculty’s perception of eLearning variables could be graded as ‘‘Good’’ (mean, 89.15; variance, 153.168; std. dev., 12.376). Cronbach’s alpha for each section was as follows: section 1 (evaluation of overall e-learning experience), 0.874; section 2 (training received), 0.940; and section 3(applying skills and knowledge in the educational process through eLearning), 0.872.

EFA on the 21-item questionnaire was 0.943, with a significance level of 0.000 with Bartlett’s test. The dimensionality of the instrument was analysed using CFA. KMO value (KMO=0.943) and Bartlett’s test of sphericity (value=10061.978, *p*<0.05) demonstrated that the raw data were suitable for the application of factor analysis ( Table 1).

**Table 1.** Kaiser-Meyer-Olkin and Bartlett’s test of the questionnaire.

Measures	Statistic
KMO measure of sampling adequacy	0.943
*Bartlett’s test of sphericity* 1. Approximate chi-squared	10061.978
2. Df	210
3. Significance	0.000

The common communalities of the instrument used are presented in Table 2; all the items had a value greater than 0.50, which indicated that the quality of the measurement was satisfactory.

**Table 2.** Communalities on the e-learning scale among IAU faculty members.

Questions	Initial	Extraction
Blackboard is easy to use	1.000	0.712
Broadcasting and recording lectures via Zoom is easy	1.000	0.415
Using Blackboard to collaborate is easy	1.000	0.533
I am not experiencing difficulties when giving tests on Blackboard	1.000	0.652
Level of satisfaction with provided technical support	1.000	0.767
It is easy to contact technical support for help	1.000	0.871
The services provided by the technical support team are sufficient	1.000	0.878
Technical support team quickly responds to my requests	1.000	0.881
How do you rate the training topics?	1.000	0.631
How would you rate the trainer in terms of their level of knowledge on the training topics?	1.000	0.755
How would you rate the trainer’s management of the sessions?	1.000	0.795
How the trainer handles participants' questions	1.000	0.753
The trainer's ability to communicate and communicate with the trainees	1.000	0.799
How do you rate the level of discussion	1.000	0.725
How would you rate your training experience using Zoom?	1.000	0.609
How do you evaluate the adequacy of the training time?	1.000	0.496
How do you rate the training materials?	1.000	0.669
The extent to which the training is consistent with my job goals	1.000	0.718
My ability to apply what I learned in the educational process	1.000	0.750
What I learned contributed to developing specific skills that can affect my success in my workplace	1.000	0.786
I recommend this training course to my colleagues.	1.000	0.666

Table 3 shows the percentage of responses for each statement. The faculty’s perception of the quality of eLearning experience at IAU was found to be high ( Table 4). A positive correlation existed between the eLearning variables that indicate an overall satisfaction with the provided services ( Table 5). The results of the factor loadings on the eLearning scale showed that all items had values greater than 0.5, which indicated that the survey’s quality was satisfactory ( Table 6).

**Table 3.** Percentage of faculty responses to e-learning variables.

S.No	Questionnaire (N=634)	Strongly disagree (%)	Disagree (%)	True sometimes (%)	Agree (%)	Strongly agree (%)
***How do you evaluate your e-learning experience (as a faculty member)?***						
**1**	Blackboard is easy to use	2 (0.3%)	16 (2.5%)	45 (7.1%)	252 (38.7%)	319 (50.3%)
**2**	Broadcasting and recording lectures via Zoom is easy	0 (0.0%))	7 (1.1%)	39 (6.2%)	212 (33.4%)	376 (59.3%)
**3**	Using Blackboard to collaborate is easy	1 (0.2%)	22 (3.5%)	206 (32.5)	195 (30.8)	210 (33.1%)
**4**	I am not experiencing difficulties when giving tests on Blackboard	22 (3.5%)	71 (11.2%)	170 (26.8%)	158 (24.9%)	213 (33.6%)
**5**	Level of satisfaction with provided technical support	3 (0.5%)	20 (3.2%)	76 (12.0%)	219 (34.5%)	316 (49.8%)
**6**	It is easy to contact technical support for help	7 (1.1%)	33 (5.2%)	123 (19.4%)	191 (30.1%)	280 (44.2%)
**7**	The services provided by the technical support team are sufficient	4 (0.6)	27 (4.3%)	115 (18.1%)	210 (33.1%)	278 (43.8%)
**8**	Technical support team quickly responds to my requests	5 (0.8%)	22 (3.5%)	135 (21.3%)	182 (28.7%)	290 (45.7%)
***Training received***						
**9**	How do you rate the training topics?	1 (0.2%)	18 (2.8%)	74 (11.7%)	209 (33.0%)	332 (52.4%)
**10**	How would you rate the trainer in terms of level of their knowledge on the training topics?	2 (0.3%)	11 (1.7%)	71 (11.2%)	210 (33.1%)	340 (53.6%)
**11**	How would you rate the trainer’s management of the session?	6 (0.9%)	24 (3.8%)	81 (12.8%)	213 (33.6%)	310 (48.9%)
**12**	How the trainer handles participants' questions	6 (0.9%)	23 (3.6%)	64 (10.1%)	199 (31.4%)	342 (53.9%)
**13**	The trainer's ability to communicate and communicate with the trainees	5 (0.8%)	20 (3.2%)	76 (12.0%)	182 (28.7%)	351 (55.4%)
**14**	How do you rate the level of discussion	11 (1.7%)	37 (5.8%)	89 (14.0%)	228 (36.0%)	269 (42.4%)
**15**	How would you rate your training experience using Zoom?	7 (1.1%)	22 (3.5%)	85 (13.4%)	181 (28.5%)	339 (53.5%)
**16**	How do you evaluate the adequacy of the training time?	11 (1.7%)	42 (6.6%)	96 (15.1%)	206 (32.5%)	279 (44.0%)
**17**	How do you rate the training materials?	3 (0.5%)	26 (4.1%)	72 (11.4%)	221 (34.9%)	312 (49.2%)
***Applying skills and knowledge in the educational process through eLearning at Imam Abdurrahman Bin Faisal*** ***University***						
**18**	The extent to which the training is consistent with my job goals	4 (0.6%)	13 (2.1%)	46 (7.3%)	271 (42.7%)	300 (47.3%)
**19**	My ability to apply what I learned in the educational process	6 (0.9%)	18 (2.8%)	55 (8.7%)	277 (43.7%)	278 (43.8%)
**20**	What I learned contributed to developing specific skills that can affect my success in my workplace	6 (0.9%)	10 (1.6%)	58 (9.1%)	227 (35.8%)	333 (52.5%)
**21**	I recommend this training course to my colleagues.	8 (1.3%)	13 (2.1%)	51 (8.0%)	186 (29.3)	376 (59.3%)
***Overall satisfaction***						
**22**	**How satisfied are you with our services**	0 (0.0%)	9 (1.4%)	79 (12.5%)	307 (48.4%)	239 (37.7%)

**Table 4.** Level of e-learning experience among faculty members at IAU.

S.No.	Questionnaire (N=634)	Mean	SD	Level
***How do you evaluate your e learning experience (as a faculty member)?***				
**1**	Blackboard is easy to use	4.37	0.752	High
**2**	Broadcasting and recording lectures via Zoom is easy	4.51	0.663	High
**3**	Using Blackboard to collaborate is easy	3.93	0.898	High
**4**	I am not experiencing difficulties when giving tests on Blackboard	3.74	1.140	High
**5**	Level of satisfaction with provided technical support	4.30	0.033	High
**6**	It is easy to contact technical support for help	4.11	0.966	High
**7**	The services provided by the technical support team are sufficient	4.15	0.908	High
**8**	Technical support team quickly responds to my requests	4.15	0.927	High
***Training***				
**9**	How do you rate the training topics?	4.35	0.806	High
**10**	How would you rate the trainer in terms of their level of knowledge on the training topics?	4.38	0.776	High
**11**	How would you rate the trainer’s management of the session?	4.26	0.888	High
**12**	How the trainer handle participants' questions	4.34	0.871	High
**13**	The trainer's ability to communicate and communicate with the trainees	4.35	0.867	High
**14**	How do you rate the level of discussion	4.12	0.971	High
**15**	How would you rate your training experience using Zoom?	4.30	0.905	High
**16**	How do you evaluate the adequacy of the training time?	4.10	1.002	High
**17**	How do you rate the training materials?	4.28	0.857	High
***Applying skills and knowledge in the educational process through eLearning at Imam Abdurrahman Bin Faisal University***				
**18**	The extent to which the training is consistent with my job goals	4.34	0.755	High
**19**	My ability to apply what I learned in the educational process	4.27	0.809	High
**20**	What I learned contributed to developing specific skills that can affect my success in my workplace	4.37	0.791	High
**21**	I recommend this training course to my colleague.	4.43	0.826	High
***Overall satisfaction with the e-learning process at IAU***				
**22**	**How satisfied are you with our services**	4.22	0.713	High

**Table 5.** Correlation between e-learning variables and overall satisfaction with our services.

	How do you evaluate your e-learning experience	Training	Skills and knowledge	Overall satisfaction with e-learning
How do you evaluate your e-learning experience	1			
Training	0.333 **	1		
Skills and knowledge	0.357 **	0.447 **	1	
Overall satisfaction with e-learning	0.354 **	0.465 **	0.622 **	1

** *Correlation is significant at the 0.01 level (2-tailed)*

**Table 6.** Results of the factor loadings on the e-learning scale among IAU faculty members.

Questions	1	2	3	4
Blackboard is easy to use	0.816			
Broadcasting and recording lectures via Zoom is easy	0.566			
Using Blackboard to collaborate is easy	0.693			
I am not experiencing difficulties when giving tests on Blackboard	0.739			
Level of satisfaction with provided technical support		0.770		
It is easy to contact technical support for help		0.86		
The services provided by the technical support team are sufficient		0.859		
Technical support team quickly responds to my requests		0.872		
How do you rate the training topics?			0.709	
How would you rate the trainer in terms of level of their knowledge on the training topics?			0.825	
How would you rate the trainer’s management of the session?			0.846	
How the trainer handles participants' questions			0.824	
The trainer's ability to communicate and communicate with the trainees			0.846	
How do you rate the level of discussion			0.799	
How would you rate your training experience using Zoom?			0.704	
How do you evaluate the adequacy of the training time?			0.627	
How do you rate the training materials?			0.696	
The extent to which the training is consistent with my job goals				0.683
My ability to apply what I learned in the educational process				0.755
What I learned contributed to developing specific skills that can affect my success in my workplace				0.788
I recommend this training course to my colleague.				0.675
Eigenvalue	9.945	2.461	1.338	1.117
Variance explained (%)	47.358	11.718	6.373	5.318
Total variance explained (%)	70.767			

**Figure 1.**
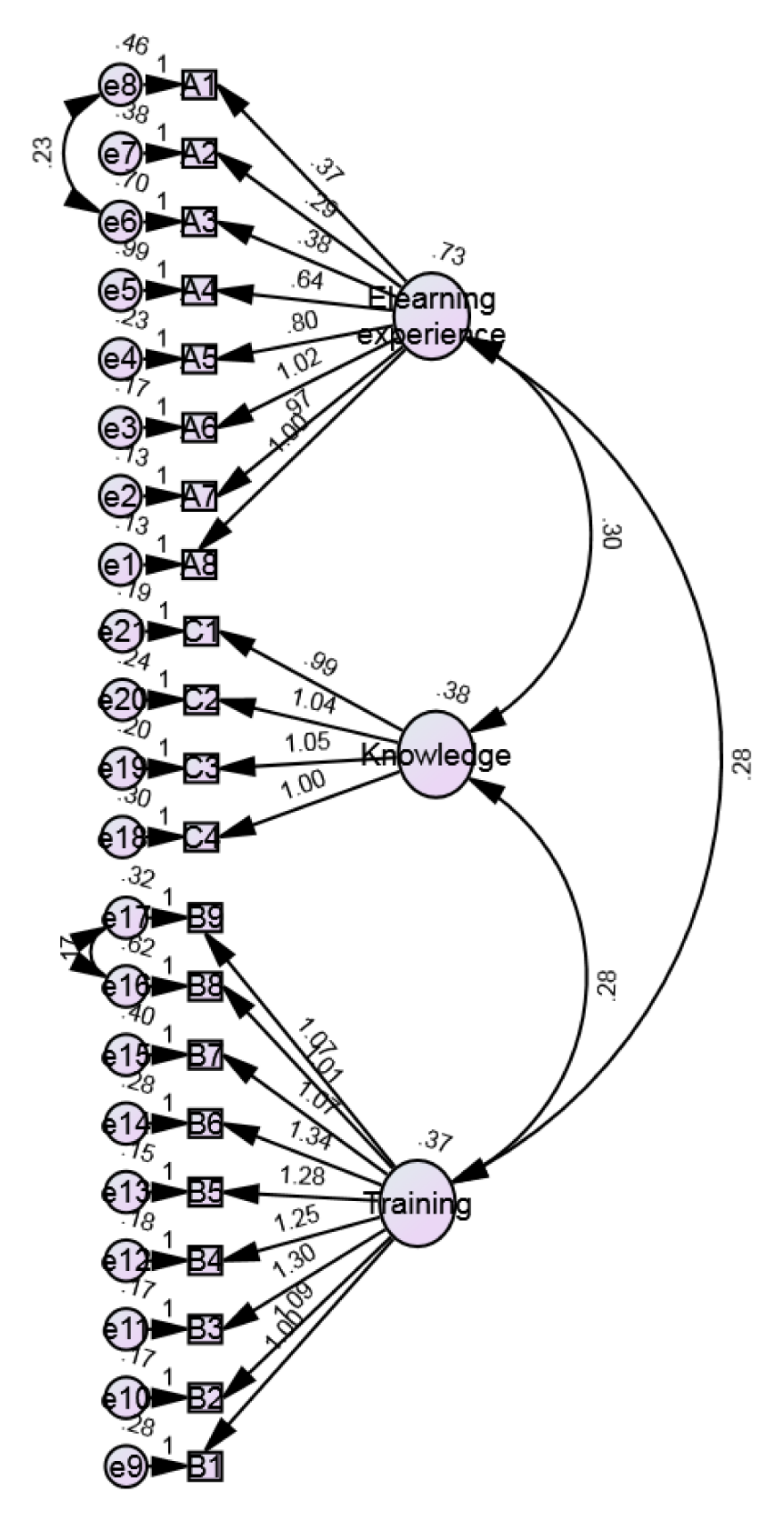
Structural equation modelling (SEM) for the eLearning evaluation of IAU faculty members following Kirkpatrick.

In this study, SEM analysis resulted in the model depicted in Figure 1, and the following characteristics: n=634, df=184, chi-squared=966.286, and p=0.000 (<0.05). Therefore, it is concluded that the proposed SEM model used in this study adequately fits the sample data representing IAU faculty members. The results of the relationship between each item and the proposed three dimensions show that the path coefficient between each item and the proposed 21-item questionnaire is positive and significant (p-value<0.05). The results show that there is a positive significant relationship between each item and the proposed three dimensions ranging from 0.290 to 1.339, which is given in Table 7. In this study, the Normed Fit Index (NFI), Relative Fit Index (RFI), Incremental Fit Index (IFI), Tucker-Lewis Index (TLI) and Comparative Fit Index (CFI) values of 0.905, 0.892, 0.922, 0.911, and 0.922, respectively, were highly consistent, suggesting that the proposed model represented an adequate fit to the data ( Table 8). The CFI=1 (>0.082) ( Table 4) and the Root Mean Square Error of Approximation (RMSEA) for the proposed model are equal to 0.0001 (p-value<0.05) ( Table 9– Table 11), which indicates that the model has a good fit. This finding is supported by a study by Bryne (2009), which stated that the NFI, RFI, IFI, TLI and CFI range from 0 to 1, with values closer to 1 being indicative of a good fit. In conclusion, SEM analysis showed that the items observed under the proposed four dimensions are acceptable to measure the experience of eLearning working by IAU faculty members during the COVID-19 outbreak.

**Table 7.** Multiple regression analysis of e-learning.

Model	R	R ^2^	Adjusted R ^2^	SE of estimation	F-value
***Overall satisfaction with the e-learning process at IAU***	0.424	0.180	0.176	0.832	0.0001 *

Note: *Significant at the 0.05 level

**Table 8.** Prediction of overall eLearning experiences at IAU.

Dimensions	Unstandardized β	Coefficients SE	Standardized coefficients β	t-value	p-value
Constant	1.409	0.242		5.827	0.0001 *
How do you evaluate your e-learning experience (as a faculty member)?	0.231	0.054	0.179	4.286	0.0001 *
Training received	0.206	0.55	0.177	3.737	0.0001 *
Applying skills and knowledge in the educational process	0.207	0.062	0.160	3.356	0.001 *

*Significant at the 0.05 level

**Table 9.** Regression weights for distance working among IAU faculty.

Variables	Path	Construct	Estimate	Standard Error	Critical Ratio	*p* value
Technical support team quickly responds to my requests	<---	E-learning experience	1.000			
The services provided by the technical support team are sufficient	<---	E-learning experience	0.974	0.025	39.493	0.0001
It is easy to contact technical support for help	<---	E-learning experience	1.023	0.027	38.169	0.0001
Level of satisfaction with provided technical support	<---	E-learning experience	0.802	0.027	29.878	0.0001
I am not experiencing difficulties when giving tests on Blackboard	<---	E-learning experience	0.644	0.049	13.160	0.0001
Using Blackboard to collaborate is easy	<---	E-learning experience	0.382	0.040	9.460	0.0001
Broadcasting and recording lectures via Zoom is easy	<---	E-learning experience	0.290	0.030	9.729	0.0001
Blackboard is easy to use	<---	E-learning experience	0.373	0.033	11.275	0.0001
How do you rate the training topics?	<---	Training	1.000			
How would you rate the trainer in terms of their level of knowledge on the training topics?	<---	Training	1.087	0.048	22.739	0.0001
How would you rate the trainer’s management of the session?	<---	Training	1.299	0.054	23.947	0.0001
How the trainer handles participants' questions	<---	Training	1.249	0.053	23.394	0.0001
The trainer's ability to communicate and communicate with the trainees	<---	Training	1.279	0.053	24.188	0.0001
How do you rate the level of discussion	<---	Training	1.339	0.060	22.332	0.0001
How would you rate your training experience using Zoom?	<---	Training	1.066	0.057	18.617	0.0001
How do you evaluate the adequacy of the training time?	<---	Training	1.015	0.064	15.746	0.0001
How do you rate the training materials?	<---	Training	1.067	0.054	19.834	0.0001
I recommend this training course to my colleague.	<---	Knowledge	1.000			
What I learned contributed to developing specific skills that can affect my success in my workplace	<---	Knowledge	1.054	0.051	20.531	0.0001
My ability to apply what I learned in the educational process	<---	Knowledge	1.043	0.053	19.852	0.0001
The extent to which the training is consistent with my job goals	<---	Knowledge	0.993	0.049	20.279	0.0001

**Table 10.** Baseline comparisons.

Model	NFI Delta1	RFI rho1	IFI Delta2	TLI rho2	CFI
Default model	**0.905**	**0.892**	**0.922**	**0.911**	**0.922**
Saturated model	1.000		1.000		1.000
Independent model	0.000	0.000	0.000	0.000	0.000

**Table 11.** Root mean square error of approximation (RMSEA).

Model	RMSEA	LO 90	HI 90	PCLOSE
Default model	**0.082**	**0.077**	**0.087**	**0.00**
Independent model	0.274	0.269	0.279	0.000

## Discussion

IAU promotes leadership qualities, encourages and supports high-end basic and applied research activities (medicine, arts and sciences, and computing), and enhances researcher skills with state-of-the-art facilities. IAU has students and faculty members from different cities and regions. During onsite/traditional classes, the chance for infection and spread is high among students due to mingling. The online management and workload assessment of faculty are critical for strategic balance ( Conceição & Lehman, 2011; Davies *et al.*, 2005). This study was conducted to evaluate eLearning variables from the perspective of IAU faculty members using a questionnaire. The questionnaire’s reliability was studied using Cronbach’s α coefficient. The criterion value was statistically studied with KMO (Kaiser-Meyer-Olkin) and Bartlett’s test. The results indicated that all the items had a value greater than 0.50, which indicated that the quality of the measurement was satisfactory.

In the first section of the questionnaire, the eLearning experience of faculty was evaluated. The faculty members were asked about their experience using Blackboard, training, and applying their learned skills and knowledge through eLearning. Bower *et al.* (2001) stated that online distance education requires effective training sessions and a change in the pedagogical approach. In addition, such a web-based teaching approach requires certain preassessment measures to ensure the validity and results ( Buchanan, 1999; Carnevale, 2004; Schifter, 2000a; Schifter, 2000b). Our results show a unanimous level of satisfaction of faculty members using the Blackboard eLearning tool. In the first instance, the ease of using Blackboard received mostly positive responses of ‘strongly agree’ (50.3%) and ‘agree’ (38.7%), indicating a higher proportion of faculty members with a strong commitment to the online working mode of action. Broadcasting and recording lectures via Zoom using Blackboard received mostly ‘strongly agree’ (59.3%) and ‘agree’ (33.4%) responses. Very few responded ‘true sometimes’ (6.2%), ‘disagree’ (1.1%) and ‘totally disagree’ (0.0%). The positive responses of respondents indicate the ease of using the Blackboard platform to provide course lessons using menu items and conducting Zoom classes with students through built content options. In the case of Blackboard collaboration (virtual classroom), the ‘true sometimes’ (32.5%) responses increased, similar to the ‘strongly agree’ (33.1%) and ‘agree’ (30.8%) responses. Impressively, the disagreement response still has a lower proportion (<4%). An increase in ‘true sometimes’ indicates that respondents have some reluctance or reservation of using Blackboard as a video tutoring platform. Conducting online tests using this software was found to be easier as most respondents positively agreed (58.5%). In total, 26.8% of respondents answered ‘true sometimes’ while few disagreed (11.2%) and strongly disagreed (3.5%). Faculty members expressed positive agreement and strong satisfaction with the provided technical support (strongly agree, 49.8%; agree, 34.5%). Less than 4% expressed disagreement, while 12% responded ‘true sometimes’. Furthermore, stronger agreement was given by faculty members for the easy contact, responses and services provided by technical support assistance.

The second section of the questionnaire was related to the experience of the training received. Cho & Berge (2002) reported that a major barrier in distance training is administrative, technical experts and the infrastructure system. However, in the present study, a strong positive response was given to training experience. Respondents were also impressed and agreed on the trainer’s knowledge expertise on training topics. Strong affirmative statements were recorded for the trainer’s session management and the way they handled participants’ questions. Similarly, the trainer’s ability to communicate with trainees and the level of discussion received strong positive responses. Training experience using the Zoom platform, training time and training materials received positive responses. For the overall training sessions, approximately 7–15% of respondents expressed the statement of ‘true sometimes’ while very few provided negative responses.

The third section of the questionnaire was related to the application of skills and knowledge in the educational process through eLearning at IAU. Substantial positive responses with 47.3% of respondents answering ‘strongly agree’ and 42.7% answering ‘agree’ indicated that the organized training was consistent with faculty’s job goals. Similarly, the faculty revealed that they were able to apply the learned experience during their educational process (agreement of 43.7% and 43.8%, respectively). A high percentage of respondents agreed that training also contributed to developing specific skills that can boost their success in the workplace and accepted that they would also promote this training course to their colleagues. Overall, the faculty members expressed satisfaction with the provided Blackboard service.

The level of perception of faculty members with respect to eLearning experience at IAU was found to be impressively high with a mean score higher than 4 ( Table 4). A positive correlation exists between eLearning variables that indicates an overall satisfaction with the provided services ( Table 5). The results of the factor loadings on the eLearning scale showed that all items had values greater than 0.5, which indicated that the survey’s result quality was satisfactory ( Table 6). The observed positive results of eLearning experience can be correlated to several IAU training initiatives offered to faculty members through the Deanship of Academic Development (DAD). Key training program approaches to online classes are classified into short training programmes, intensive training programmes and material resource support. The professional development training programme involves improving competency in teaching/learning, lecture preparations and mentorship training programmes. Training topics are based on assessment, surveys, reports to the Deanship of Quality and Accreditation (DQAA), student course evaluations, faculty, academic program evaluations, benchmarking teaching and learning practices, trainer questionnaires, and DAD forum recommendations. In addition, the training content materials were updated in the training portal on Blackboard and IAU website ( DAD, 2020).

Mainly, the key strategy points focus on faculty online communication skills, leadership skills, conceptual thinking, learning as a team, teaching in a creative way, interpersonal student communication skills, deep learning, lecture planning, an artistic teaching approach and class management.

A faculty professional development series was conducted by IAU. The topics was related on utilizing educational technology and teaching methods. The framework includes theoretical backed interactive sessions, using technological tools to improve student engagement, motivating the students by improving the learning environment and intellectual concept activities, improving competency and fluency in English, microteaching (teaching through practice), metacognition (higher-order thinking), effective questioning strategies, avoiding common teaching mistakes, flipped classrooms (instructional strategy), knowing students’ learning styles and welcoming students on the first day of class.

The professional training for faculty also includes improving effective assessment and evaluation skills. The module covers the different types of concepts, methods, types and concepts based on assessment. Increasing questioning, thinking skills, teaching strategies and question paper setting tend to improve higher-order thinking capabilities. Faculty members are guided to use performance-based assessment and portfolios; improve the capability of analysing test results; establish question items to motivate higher-order thinking; assess project work and lab-based learning; and improve soft skills such as emotional intelligence, team-based work, interactions, metacognition skills and leadership. Furthermore, faculty members are trained on grading practices, effective rubrics and constructive feedback.

Importantly, a survey finding by Bonk (2002) stated that faculty members should be trained for online teaching in the online world. Considering such recommendations, the development series focused on mentoring benefits, improving oral communications, and 21 ^st^ century skills (collaboration, critical thinking, communications, creativity, and emotional intelligence) in higher education. Training includes assessing new learning and teaching strategies, similar to the COVID-19 pandemic situation; and how to publish in journals related to education. The module comprises observation of classroom behaviours and interactions and the promotion of learning through activities. Faculty members were taught strategies for formulating key principles of critical thinking and student engagement. The adult learning concept and principles were taught to be applied in knowledge transfer from classroom to actual work settings, as seen in the current pandemic situation. The concept of self-efficacy from the perspective of faculty and department was the focus. Similarly, preventing faculty burnout during adverse situations such as COVID-19 and different strategies to overcome faculty burnout are taught.

SEM was used to evaluate the experience of eLearning at IAU. This model has been effectively used to analyse the structural variables in educational-based research ( Jansson *et al.*, 2019). Based on the survey study, a model was constructed using SEM analysis ( Figure 1). The SEM study showed that items studied under the proposed three dimensions are acceptable for measuring the eLearning experience of IAU faculty members during the COVID-19 pandemic. Overall, the modules were found to be effective in the present situation and able to continue the practice of teaching and learning in the online mode of action. The expressed eLearning satisfaction level by faculty and online trainings adopted by IAU can be an effective strategy to combat online teaching challenges. 

## Conclusion

The COVID-19 pandemic presents an unprecedented challenge in higher education. This study explored faculty readiness for online teaching during the COVID-19 crisis at IAU. The survey responses by the faculty indicate their high satisfaction using eLearning tools. The Blackboard online teaching software tool, recording lectures using the Zoom platform, virtual classrooms and online tests received strong positive responses. Faculty responded positively to the technical and training support rendered by IAU. Overall, the study found that the eLearning training and modules provided by IAU were effective in the present pandemic situation.

## Data availability

### Underlying data

Figshare: E-learning datasheet of Faculty readiness for online teaching at Imam Abdulrahman Bin Faisal University during the COVID-19 crisis: a cross-sectional study, https://doi.org/10.6084/m9.figshare.14406434 ( Almahasheer *et al.*, 2021).

Data are available under the terms of the Creative Commons Attribution 4.0 International license (CC-BY 4.0).
