# Supplementary material for: Faculty readiness for online teaching at Imam Abdulrahman Bin Faisal University during the COVID-19 crisis: a cross-sectional study
Source: F1000Res. 2022 Jul 28;10:840. Originally published 2021 Aug 20. [Version 3] doi: 10.12688/f1000research.28173.3 (PMC10911066; doi:10.12688/f1000research.28173.3)
Supplement: Peer review history

# Reviewer report: Reviewer response for version 3

**DOI:** 10.5256/f1000research.134554.r247856

**Published:** 2024-02-28

**Authors:** Rewanee Chaichaowarat

**Affiliations:** 1 Udon Thani Rajabhat University, Udon Thani, Udon Thani, Thailand

Thank you for affording me the opportunity to review your research article. I posit that your findings hold potential significance for faculty members worldwide, offering insights into the challenges faced by educators and university students amidst the Covid-19 pandemic. However, I observe that your article requires further refinement.

 In the Introduction section, it is advisable for the author to incorporate more recent scholarly resources pertaining to remote learning, online learning (both synchronous and asynchronous), blended learning, and offline modalities. Over the past 2-3 years, particularly due to widespread school closures and the consequent transition to online pedagogy prompted by the Covid-19 crisis, a plethora of studies exploring this domain have emerged. The author should prioritize recent literature in selecting and referencing sources for the article.

 Regarding the Methods section, while the authors mention employing an exploratory study design with a questionnaire developed through four brainstorming sessions with higher education experts and faculty members, there is a lack of clarity regarding the methodology employed in this process, the number of experts involved, and the process of finalizing the questionnaire's validity. Additionally, the inclusion of a separate literature review section and a structured discussion on relevant sources pertaining to the questionnaire's structure is warranted.

 Furthermore, the information provided in the Results section necessitates better integration with the data collection process. Specifically, the reliability and validity of the instrument should be elucidated within the context of questionnaire creation.

 The purpose statement of the article remains somewhat obscure. While the introduction alludes to the aim of analyzing the significant role of digital systems and evaluating the readiness of IAU faculty members to transition to online teaching during Covid-19 through survey-based methods, the Methods section indicates the utilization of an exploratory study design to predict the characteristics and management of e-learning advantages by faculty members. Clarification of the article's overarching purpose, especially if it represents only a segment of a broader study, would enhance reader comprehension.

 I am of the opinion that your article holds substantial practical relevance. It stands to aid faculty members in gaining a deeper understanding of digital systems and assessing the preparedness required for transitioning to online teaching amid the challenges posed by COVID-19. Nevertheless, there are certain aspects of the Results section that warrant reconsideration in alignment with the study's objectives. It would be beneficial for readers if the significance of digital systems and the readiness of IAU faculty members for online teaching during the pandemic were more thoroughly explained and illustrated within the context of the study's aims.

Is the work clearly and accurately presented and does it cite the current literature?

Partly

If applicable, is the statistical analysis and its interpretation appropriate?

Partly

Are all the source data underlying the results available to ensure full reproducibility?

Partly

Is the study design appropriate and is the work technically sound?

Yes

Are the conclusions drawn adequately supported by the results?

Partly

Are sufficient details of methods and analysis provided to allow replication by others?

Partly

Reviewer Expertise:

Teacher Education, Teaching and Learning, Curriculum Development, Design Thinking for education,  Professional Learning Community (PLC)

I confirm that I have read this submission and believe that I have an appropriate level of expertise to confirm that it is of an acceptable scientific standard, however I have significant reservations, as outlined above.

## References

[1] : Teaching and Learning during the COVID-19 Pandemic: Attributes and Readiness of Thai Teachers. *The Clearing House: A Journal of Educational Strategies, Issues and Ideas* .2023;96(2) : 10.1080/00098655.2022.2163970 70-78 10.1080/00098655.2022.2163970

# Reviewer report: Reviewer response for version 2

**DOI:** 10.5256/f1000research.80828.r120658

**Published:** 2022-02-10

**Authors:** Martin Daumiller

**Affiliations:** 1 Department of Psychology, University of Augsburg, Augsburg, Germany

Overall, I have found that this manuscript makes a relevant contribution to the literature and is well written. However, there are still issues that warrant attention:

1. The introduction on COVID-19 is very lengthy, yet in the theoretical background only little is written about how COVID-19 affected faculty (e.g., Daumiller *et al. *(2021) ^1^, Delivering High-Quality Instruction Online in Response to COVID-19: Faculty Playbook, Valsaraj *et al.* (2021) ^2^, and related works). Yet, such information would be highly relevant for the outset of the article.
2. A contemporary understanding of "validity" should be used. See AERA, APA, NCME recommendations, according to which a questionnaire itself cannot have "construct validity".
3. In the method section, the authors state they used a CFA, but later findings of a EFA are described. This is inconsistent. What are the fit indices of the CFA and its results? A CFA would appear more appropriate given that an established scale is used, and in order to prepare the SEM.
4. Which cut off values are used to determine adequate model fit? The fit indices fall short of many commonly used criteria (e.g., CFI, TLI > .95), and partly even more lax recommendations (e.g. CFI, TLI > .90).
5. What do you mean that CFI and RMSEA are equal? This seems impossible, given that high CFI values and low RMSEA represent good model fit. Why would it be necessary to test them for equivalence?
6. The information presented in the tables and figures is partly redundant. I suggest to only present them either in the figure or in the respective table.
7. It would be good to illustrate the SEM as a figure so that it becomes clear how this model looked like. Similar to the results of the CFA in Figure 1.
8. The authors should also add their analytical script to the dataset so that researchers can reproduce their results.

Is the work clearly and accurately presented and does it cite the current literature?

Partly

If applicable, is the statistical analysis and its interpretation appropriate?

Partly

Are all the source data underlying the results available to ensure full reproducibility?

No

Is the study design appropriate and is the work technically sound?

Yes

Are the conclusions drawn adequately supported by the results?

Partly

Are sufficient details of methods and analysis provided to allow replication by others?

No

Reviewer Expertise:

Faculty motivation, experiences, and only teaching; structural equation modelling

I confirm that I have read this submission and believe that I have an appropriate level of expertise to confirm that it is of an acceptable scientific standard, however I have significant reservations, as outlined above.

## References

[1] : Shifting from face-to-face to online teaching during COVID-19: The role of university faculty achievement goals for attitudes towards this sudden change, and their relevance for burnout/engagement and student evaluations of teaching quality. *Computers in Human Behavior* .2021;118: 10.1016/j.chb.2020.106677 10.1016/j.chb.2020.106677 PMC975800136570330

[2] : Faculty experiences on emergency remote teaching during COVID-19: a multicentre qualitative analysis. *Interactive Technology and Smart Education* .2021;18(3) : 10.1108/ITSE-09-2020-0198 319-344 10.1108/ITSE-09-2020-0198

# Reviewer report: Reviewer response for version 2

**DOI:** 10.5256/f1000research.80828.r120632

**Published:** 2022-01-25

**Authors:** Shahul Hameed Pakkir Mohamed

**Affiliations:** 1 Department of Physical Therapy, Faculty of Applied Medical Sciences, University of Tabuk, Tabuk, Saudi Arabia

Thank you very much for your responses. All the comments were addressed in the manuscript.

Is the work clearly and accurately presented and does it cite the current literature?

Partly

If applicable, is the statistical analysis and its interpretation appropriate?

Partly

Are all the source data underlying the results available to ensure full reproducibility?

Partly

Is the study design appropriate and is the work technically sound?

Partly

Are the conclusions drawn adequately supported by the results?

Partly

Are sufficient details of methods and analysis provided to allow replication by others?

Partly

Reviewer Expertise:

Musculoskeletal physical therapy

I confirm that I have read this submission and believe that I have an appropriate level of expertise to confirm that it is of an acceptable scientific standard.

# Reviewer report: Reviewer response for version 1

**DOI:** 10.5256/f1000research.31164.r92444

**Published:** 2021-10-04

**Authors:** Shahul Hameed Pakkir Mohamed

**Affiliations:** 1 Department of Physical Therapy, Faculty of Applied Medical Sciences, University of Tabuk, Tabuk, Saudi Arabia

**Comments on: Faculty readiness for online teaching at IAU during the COVID-19 crisis.**

1. The demographic details are missing in the table and in the interpretation of the results?
2. It is better to add a table relationship between demographic data (such as academic rank, age, years of teaching experience, attendance of training courses, etc...) of faculty members and their e-learning experience, received training information, and applied skills and knowledge?
3. What type of sampling was used in this study?
4. What are the challenges or barriers the faculty members at the IAU have met while using Blackboard?
5. What are the limitations, suggestions, and recommendations in the study?

Is the work clearly and accurately presented and does it cite the current literature?

Partly

If applicable, is the statistical analysis and its interpretation appropriate?

Partly

Are all the source data underlying the results available to ensure full reproducibility?

Partly

Is the study design appropriate and is the work technically sound?

Partly

Are the conclusions drawn adequately supported by the results?

Partly

Are sufficient details of methods and analysis provided to allow replication by others?

Partly

Reviewer Expertise:

Musculoskeletal physical therapy

I confirm that I have read this submission and believe that I have an appropriate level of expertise to confirm that it is of an acceptable scientific standard, however I have significant reservations, as outlined above.

# Response

**Published:** 2021-12-10

**Authors:** Nuhad Abdullah Alomair

**Affiliations:** Imam Abdulrahman BinFaisal University, Saudi Arabia

- **The demographic details are missing in the table and in the interpretation of the results?**

**Response:**

 The aim of the study is to analyse only the significant role of the digital system and evaluate the readiness of IAU faculty members to transition to online teaching during COVID-19 using survey-based methods. Hence, the authors have not collected the demographic data of the IAU faculty members. However, the authors stated that the demographic data of faculty members can be included in further research in the Limitations and Recommendations Section.

**Limitations and Recommendations**

 This study is restricted to the faculty members of a single public university, and future work can be conducted with the enclosure of all Saudi universities. **In further research, the demographic data of faculty members can be included. **Further, ****the relationship between demographic variables of faculty members (such as age, gender, nationality, highest educational qualification, current designation, and work experience) and their e-learning experience, training, and applying skills and knowledge in the educational process through e-learning can be revealed in future studies.**** Besides, the difference in overall satisfaction among faculty members concerning their demographic variables can also be studied. It is suggested to study the influence of demographic variables on overall faculty satisfaction with the e-learning process at their respective universities. A qualitative or quantitative research can be further carried out with Saudi faculty members to reveal the challenges or barriers facing faculty members while using Blackboard. This study also recommends that higher education administrators frame and execute uniform regulations to improve teaching faculty’s satisfaction with Blackboard.

- **It is better to add a table relationship between demographic data (such as academic rank, age, years of teaching experience, attendance of training courses, etc...) of faculty members and their e-learning experience, received training information, and applied skills and knowledge?**

**Response:**

 The aim of the study is to to analyse the significant role of the digital system and evaluate the readiness of IAU faculty members to transition to online teaching during COVID-19 using survey-based methods. Hence, the authors did not analyze the relationship between demographic data of faculty members and their e-learning experience, received training information, and applied skills and knowledge. However, the authors stated that the relationship between demographic data of faculty members and their e-learning experience, received training information, and applied skills and knowledge can be revealed in future studies in the Limitations and Recommendations Section.

 The description is provided as follows:

**Limitations and Recommendations**

 This study is restricted to the faculty members of a single public university, and future work can be conducted with the enclosure of all Saudi universities. In further research, the demographic data of faculty members can be included. **Further,** **the relationship between demographic variables of faculty members (such as age, gender, nationality, highest educational qualification, current designation, and work experience) and their e-learning experience, training, and applying skills and knowledge in the educational process through e-learning can be revealed in future studies.** Besides, the difference in overall satisfaction among faculty members concerning their demographic variables can also be studied. It is suggested to study the influence of demographic variables on overall faculty satisfaction with the e-learning process at their respective universities. A qualitative or quantitative research can be further carried out with Saudi faculty members to reveal the challenges or barriers facing faculty members while using Blackboard. This study also recommends that higher education administrators frame and execute uniform regulations to improve teaching faculty’s satisfaction with Blackboard.

- **What type of sampling was used in this study?**

**Response:**

 In this study, convenience sampling technique was applied.

 The description is provided in the Methods section as follows:

**Methods**

**Participants**

 This study was conducted at Imam Abdulrahman Bin Faisal University (IAU), which is located in the Eastern Region of Saudi Arabia. A convenience sampling was used in this study. All faculty members (N=2227) of IAU who were involved in online teaching during the COVID-19 crisis in the 2019–2020 academic year were considered the population of this study, as only these faculty members used and experienced IAU e-learning facilities.

- **What are the challenges or barriers the faculty members at the IAU have met while using Blackboard?**

**Response:**

 The aim of the study is to analyse the significant role of the digital system and evaluate the readiness of IAU faculty members to transition to online teaching during COVID-19 using survey-based methods. Hence, the authors did not reveal the challenges or barriers the faculty members at the IAU have met while using Blackboard. This valuable point of the reviewer has been included in the Limitations and Recommendations Section for further research. The description is provided as follows:

**Limitations and Recommendations**

 This study is restricted to the faculty members of a single public university, and future work can be conducted with the enclosure of all Saudi universities. In further research, the demographic data of faculty members can be included. Further, **the relationship between demographic variables of faculty members (such as age, gender, nationality, highest educational qualification, current designation, and work experience) and their e-learning experience, training, and applying skills and knowledge in the educational process through e-learning can be revealed in future studies.** Besides, the difference in overall satisfaction among faculty members concerning their demographic variables can also be studied. It is suggested to study the influence of demographic variables on overall faculty satisfaction with the e-learning process at their respective universities. **A qualitative or quantitative research can be further carried out with Saudi faculty members to reveal the challenges or barriers facing faculty members while using Blackboard.** This study also recommends that higher education administrators frame and execute uniform regulations to improve teaching faculty’s satisfaction with Blackboard.

- **What are the limitations, suggestions, and recommendations in the study?**

**Response:**

 As advised, the authors have included the Limitations and Recommendations Section in the manuscript. The description provided in the manuscript is given below:

**Limitations and Recommendations**

 This study is restricted to the faculty members of a single public university, and future work can be conducted with the enclosure of all Saudi universities. In further research, the demographic data of faculty members can be included. Further, ****the relationship between demographic variables of faculty members (such as age, gender, nationality, highest educational qualification, current designation, and work experience) and their e-learning experience, training, and applying skills and knowledge in the educational process through e-learning can be revealed in future studies.** **Besides, the difference in overall satisfaction among faculty members concerning their demographic variables can also be studied. It is suggested to study the influence of demographic variables on overall faculty satisfaction with the e-learning process at their respective universities. A qualitative or quantitative research can be further carried out with Saudi faculty members to reveal the challenges or barriers facing faculty members while using Blackboard. This study also recommends that higher education administrators frame and execute uniform regulations to improve teaching faculty’s satisfaction with Blackboard.
